# A Comparative Transcriptional Landscape of Two Castor Cultivars Obtained by Single-Molecule Sequencing Comparative Analysis

**DOI:** 10.3389/fgene.2021.749340

**Published:** 2021-10-18

**Authors:** Yaxing Zhou, Guoli Zhu, Yun Wang, Zhibiao He, Wei Zhou

**Affiliations:** ^1^ Agricultural College of Inner Mongolia Minzu University, Tongliao, China; ^2^ Tongliao Academy of Agricultural Science, Tongliao, China

**Keywords:** Lm type female strains, normal amphiprotic strains, pacbio, comparative transcriptome, isoform, full-length transcriptome

## Abstract

**Background and Objectives:** Castor (*Ricinus communis* L.) is an important non-edible oilseed crop. Lm-type female strains and normal amphiprotic strains are important castor cultivars, and are mainly different in their inflorescence structures and leaf shapes. To better understand the mechanisms underlying these differences at the molecular level, we performed a comparative transcriptional analysis.

**Materials and Methods:** Full-length transcriptome sequencing and short-read RNA sequencing were employed.

**Results:** A total of 76,068 and 44,223 non-redundant transcripts were obtained from high-quality transcripts of Lm-type female strains and normal amphiprotic strains, respectively. In Lm-type female strains and normal amphiprotic strains, 51,613 and 20,152 alternative splicing events were found, respectively. There were 13,239 transcription factors identified from the full-length transcriptomes. Comparative analysis showed a great variety of gene expression of common and unique transcription factors between the two cultivars. Meanwhile, a functional analysis of the isoforms was conducted. The full-length sequences were used as a reference genome, and a short-read RNA sequencing analysis was performed to conduct differential gene analysis. Furthermore, the function of DEGs were performed to annotation analysis.

**Conclusion:** The results revealed considerable differences and expression diversity between the two cultivars, well beyond what was reported in previous studies and likely reflecting the differences in architecture between these two cultivars.

## Introduction

The castor plant (*Ricinus communis* L.), which originated in Africa, is an annual or perennial dicotyledonous. High ricinoleic acid content (80–90%) and high fatty acid content (more than 45%) in its seed oil make it one of the most important non-edible oilseed crops, and this has attracted much attention from chemists, biologists, and medical scientists (Fan et al., 2019). The inflorescence of common castor plants is gradient monoecious raceme, with male flowers on the lower portion and female flowers at the apex (Tan et al., 2015). Pistillate (bearing only female flowers) variations are bred to improve the seed yield. The Lm type castor is such a variety, obtained by exposing castor seeds to ^60^Coγ.

With the development of sequencing technology, next-generation sequencing (NGS) has become an essential method for the study of genomes, epigenomes, and transcriptomes (Fan et al., 2019). The NGS method has been used in many model and non-model plant species, and large-scale genome sequences and transcriptome data have been produced for deep analysis (Fan et al., 2019). However, the deficiencies of NGS, such as short reads, result in incompletely assembled transcripts that limit the better understanding of the transcriptomic data (Fan et al., 2019). The PacBio platform is based on the single-molecule real-time (SMRT) sequencing technology and provides longer and full-length transcripts without assembly, and can provide better information to understand the full-length transcriptome, such as alternative splicing, fusion transcripts, alternative polyadenylation, novel genes, and non-coding RNAs (Fan et al., 2019).

To gain an insight into how sex is differentially regulated at the molecular level, in the present study, full-length transcriptomes of Lm-type and normal castor cultivars were analyzed, and short-read RNA sequencing and single-molecule long-read sequencing were utilized to identify the differentially expressed genes and alternative splicing events between Lm-type female strains and normal amphiprotic strains. Furthermore, the study will provide valuable data for future studies of sex determination on castor plants.

## Materials and Methods

**FIGURE 1:**
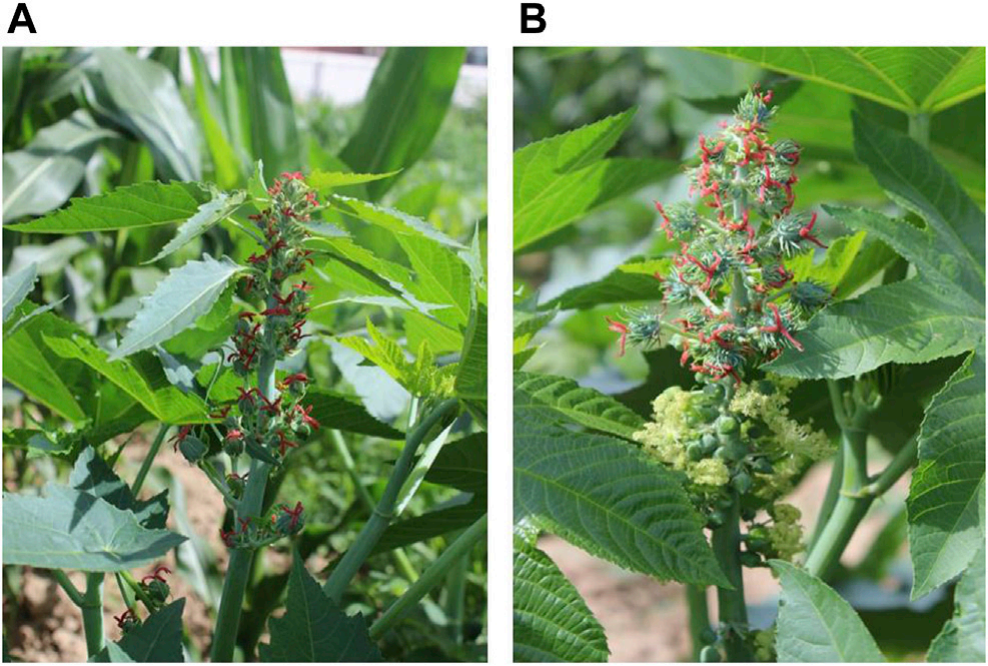
The castor plant R. communis L. **(A)** Lm-type female strains with willow-shaped leaves. **(B)** Normal amphiprotic strains.

Two cultivars, Lm-type female plants with willow-shaped leaves and normal amphiprotic plants (Figure 1), were grown at the Experimental Base of the Agricultural College of INNER MONGOLIA MINZU UNIVERSITY, Tongliao City, Inner Mongolia Autonomous Region. The geographical position is between 42°15′-45°41′ north latitude and 119°15′-123°43′ east longitude. Ten plants of each cultivar were selected when the functional leaves grew to 1–6 cm (Lm-type) or 2–15 cm (normal type) on August 2nd, 2018. The Lm-type female plants and normal amphiprotic plants were designated as F01 and F02, respectively. For each cultivar around 10g of leaves and flowers were collected and frozen in liquid nitrogen and then stored at −80°C for subsequent RNA isolation. Using the Illumina HiSeq X Ten platform, RNA was extracted from 5g of frozen leaves or flowers with two repeats, and an RNA-Seq library construction was performed following the instructions (Podnar et al., 2014).

### PacBio Library Construction and Sequencing

The total RNA was extracted from 5g mixtures of leaves and flowers (two repeats). Poly(T) oligo-attached magnetic beads (Dynal) were used to purify mRNA from about 3 µg total RNA. According to the protocols of the PacBio RS II platform, cDNA was synthesized using the SMART PCR cDNA Synthesis Kit (Clontech, CA, United States), and then fractionated with BluePippin^®^ (Sage Science, Beverly, MA, United States). Then the final libraries were constructed using the Pacific Biosciences DNA Template Prep Kit (version 2.0). SMRT sequencing was performed with the Pacific Biosciences’ real-time sequencer using C2 sequencing reagents.

### Preprocessing of SMRT Reads

The subreads were filtered using the standard protocol of the SMRT Analysis software suite (http://www.pacificbiosciences.com), and the reads of insert (ROIs) were obtained. After examining the poly(A) signals and 5′ and 3’ adaptors, full-length (FL) and non-full-length (nFL) reads were identified.

Consensus sequences were obtained from high-quality isoform sequences. The final transcriptome isoform sequences were filtered by removing the redundant sequences with the CD-HIT package (http://weizhong-lab.ucsd.edu/cdhit_suite/cgi-bin/index.cgi?cmd=cd-hit) to cluster and compare protein or nucleotide sequences.

### Alternative Splicing Analysis of Transcriptomes

To identify alternative splicing (AS) events, SpliceGrapher (Rogers et al., 2012) was used to analyze the transcriptome-wide AS events. AS events were predicted from non-redundant transcripts. The prediction criterion is as following: the sequence should be greater than 1,000 bp, the AS gap should be greater than 100 bp and at least 100 bp from the 3'-/5'-end, and there should be a 5-bp overlap in the spliced transcript. Compared with the reference castor genome (http://castorbean.jcvi.org/), the full-length transcripts can be classified as derivations from the known genes and novel genetic loci.

Candidate coding regions were identified by TransDecoder (Broad Institute, Cambridge, MA, United States) from the final transcriptome isoform sequence. Sequences were searched using BLASTX (Buchfink et al., 2015) against the NCBI non-redundant protein and the UniProt with E-value cutoff at 1 × 10^−6^. To further distinguish protein-coding and non-coding RNAs, the dbHT-Trans tool (v1.0) (Deng and Chen 2016) was used for all PacBio transcripts.

The gene ontology (GO) enrichments were analyzed using the GOseq (Young et al., 2010). The KEGG (http://www.genome.jp/kegg/) pathway analysis was implemented as reported (Kanehisa et al., 2017).

### Short-Read RNA Sequencing Analysis and Quantification of Gene Expression

The clean reads were screened from raw sequencing reads by removing low-quality reads and reads containing adaptors or ploy-Ns. Sequences of clean reads were aligned to the full-length sequences. Differential expression analysis was performed with EBSeq package (Leng et al., 2013), with FDR <0.05 and |log2 (fold-change) | ≥1.

## Results

### PacBio Iso-Seq Sequencing

The SMRT sequencing generated 456,994 polymerase reads in total, and 26.25 Gb and 16.38 Gb clean reads were obtained from Lm-type female and normal castor cultivars, respectively. Under the conditions of full passes of ≥0 and quality of >0.80, 647,205 and 328,497 ROIs were obtained from two cultivars, respectively (Supplementary Table S1). In addition, 448,217 and 258,645 full-length non-chimeric sequences were identified from Lm-type and normal castors, respectively (Supplementary Table S2).

The SMRT sequencing generated 456,994 polymerase reads in total, and 26.25 Gb and 16.38 Gb of clean reads were obtained from Lm-type female and normal castor cultivars, respectively. Under the conditions of full passes of ≥0 and quality of >0.80, 647,205 and 328,497 ROIs were obtained from two cultivars, respectively (Supplementary Table S1). In addition, 448,217 and 258,645 full-length non-chimeric sequences were identified from Lm-type and normal castors, respectively (Supplementary Table S2).

The lengths of full-length cDNA in the Lm-type female strain ranged from 281 to 11,430 bp with an average length of 2,702 bp. For the normal castor strain, the full-length cDNA showed an average length of 2,192 bp, and ranged from 303 to 9,681 bp. The N50 values of that cDNA were 3,093 and 2,408 bp in Lm-type and normal castor cultivars, respectively. Then, from 223,929 (Lm-type female cultivar) and 138,066 (normal castor) full-length consensuses cDNA, 76,068 out of 154,517 (49%) and 44,223 out of 105,536 (42%) high quality full-length consensuses were obtained, respectively. The ICE clustering results are shown in Table 1.

**TABLE 1:** The statistic results of ICE clustering.

Samples	Number of consensus isoforms	Average consensus isoforms read length	Number of polished high-quality isoforms	Number of polished low-quality isoforms	Percent of polished high-quality isoforms (%)
F01	223,929	2,552	154,517	68,417	69.00
F02	138,066	2,056	105,536	32,086	76.44

### Alternative Splicing and Polyadenylation

**FIGURE 2:**
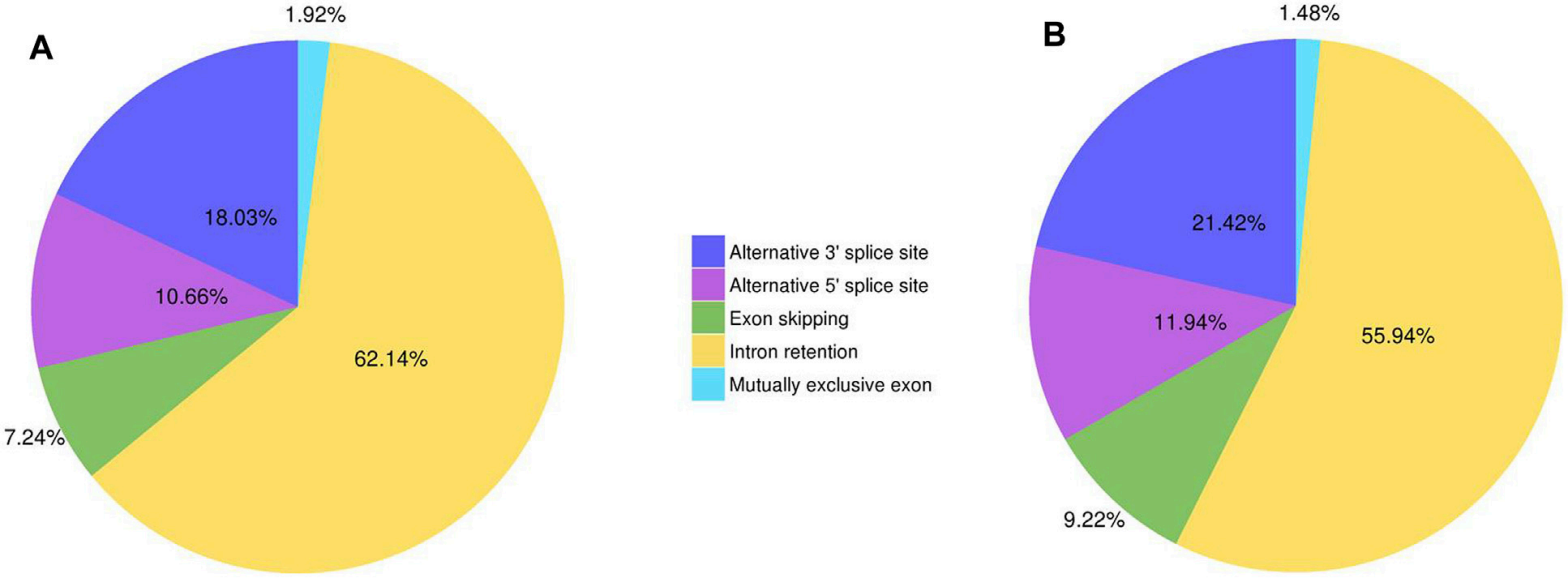
The statistics of different AS events of two cultivars. **(A)** AS events of Lm-type female strain. **(B)** AS events of normal castor.

A total of 51,613 and 20,152 AS events were found in Lm-type and normal castor cultivars, respectively, including exon skipping (ES), intron retention (IR), alternative 3′ sites (Alt. 3′), alternative 5′ sites (Alt. 5’), and mutually exclusive exons. The results showed that intron retention (IR) was the foremost AS event, with 62.14% and 55.94% in Lm-type and normal castor cultivars, respectively. The results of statistical analysis of different AS events in Lm-type female strain and normal castor were showed in Figure 2 and Supplementary Table S3.

### Comparative Analysis of LncRNA and Transcription Factors

**FIGURE 3:**
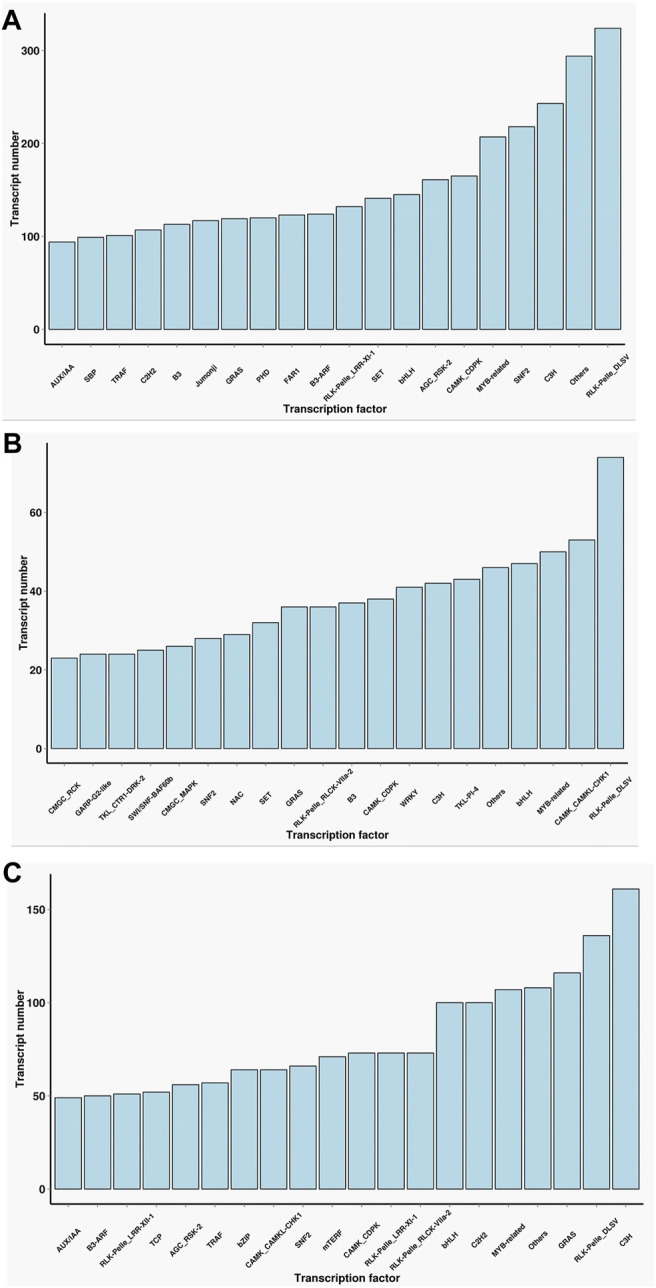
Statistics of transcription factors of two cultivars. **(A)** The number of transcription factors only in F01. **(B)** The number of transcription factors only in F01. **(C)** The number of transcription factors common in F01 and F02.

Transcription factors which need to specifically bind to certain genes are essential for the regulation of gene expression. A total of 13,239 encoded transcription factors were identified from the full-length transcriptome in the two cultivars. Furthermore, we performed a comparative analysis of the common and unique transcription factors in the two cultivars. The main transcription factor types in Lm-type female castor include Rlk-Pelle-Dlsv, C3H, SNF2, and MYB-related families. In normal castor, the dominant transcription factors were Rlk-pelle-dlsv, camk-camkl-chk1, and MYB-related bHLH types. Although the two cultivars shared some types of transcription factors, the expression of corresponding genes was completely different (Figure 3).

**FIGURE 4:**
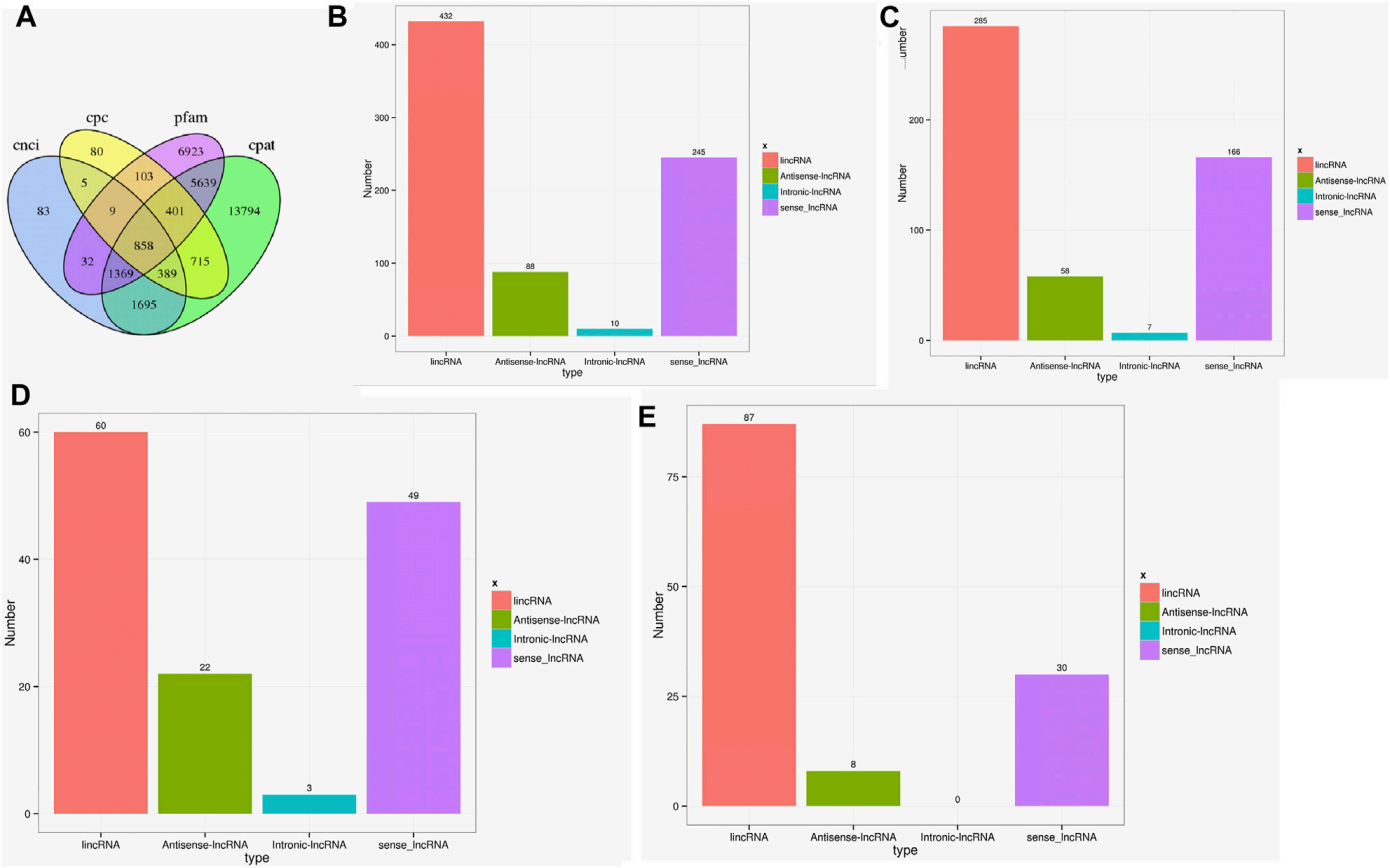
Statistics of LncRNA in two cultivars. **(A)** Venn diagram of LncRNAs using CPC, CNCI, CPAT, and PFAM software. **(B)** Statistics of LncRNA types in genomic distributions. **(C)** Statistics of LncRNA types only in F01. **(D)** Statistics of LncRNA types only in F02. **(E)** Statistics of LncRNA types common in F01 and F02.

As the key regulators in biological processes, long non-coding RNA (LncRNA) is a type of RNA that does not encode proteins (Jathar et al., 2017). A total of 858 lncRNAs were found in the two cultivars using CPC, CNCI, CPAT, and PFAM software (Gong et al., 2017). The genomic distributions of LncRNAs were classified into four types, namely lincRNA, antisense-lncRNA, intronic-lncRNA, and sense_lncRNA. The ratios of different types varied greatly, with 285 lincRNA, 58 antisense-lncRNA, 7 intronic-lncRNA, and 166 sense_lncRNA in the Lm-type, and 60, 22, 3, and 49 in the normal castor cultivar, respectively (Figure 4).

### Functional Annotation and Analysis of Isoform

**FIGURE 5:**
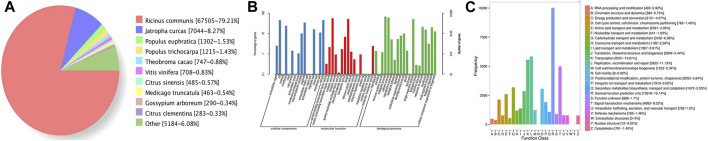
Statistics of the gene annotation in castor R. communis. **(A)** Nr homologous species distribution statistics. **(B)** GO annotation classification statistics. **(C)** COG annotation classification statistics.

For the functional annotation of gene isoforms, these genes were searched against the Genbank NR, Swissprot, GO, COG, KOG, Pfam, and KEGG databases, and a total of 85,322 genes were annotated by those seven databases. Among them, 85,286 genes (99.96%) were aligned to the NR, and 62,336 genes were matched to the SWISS-PROT (Table 2). Approximately 79.21% of genes were aligned to *R. communis*, followed by *Jatropha curcas* (8.27%) (Figure 5A).

**TABLE 2:** Statistics of annotations for genes of castor.

Anno database	Annotated number
COG Annotation	35,743
GO Annotation	61,390
KEGG Annotation	39,598
KOG Annotation	57,228
Pfam Annotation	63,089
Swissprot Annotation	62,336
eggNOG Annotation	83,826
Nr Annotation	85,286
All Annotated	85,322

The GO annotation system is a directed acyclic graph, including three categories: biological process (BP), molecular function (MF), and cellular component (CC). In this study, GO analysis was conducted using Blast2GO, and detailed GO distributions in GO categories are shown in Figure 5B. The vast majority of the genes were in cells or cell parts in the cellular component. In the molecular function class, most of the genes were classified as catalytic activity and binding. In the biological process class, genes classified as metabolic processes and cellular processes were the most common. “COG” refers to clusters of orthologous groups for eukaryotic complete genomes, and every protein in the database is assumed to be evolved from a common ancestor protein. In total, 35,743 out of 85,286 genes were classified into 25 different COG categories (Figure 5C), and the genes with general functions were the largest category, followed by replication, recombination and repair, and transcription.

The KEGG database is used to determine whether the genes are involved in specific metabolic or signal transduction pathways. In this study, a total of 125 KEGG pathways were identified. Several enriched pathways were involved in plant hormone signal transduction (ko04075), starch and sucrose metabolism (ko00500), and protein processing in the endoplasmic reticulum (ko04141) (Supplementary list S1).

### Functional Comparative Analysis of Isoform

**FIGURE 6:**
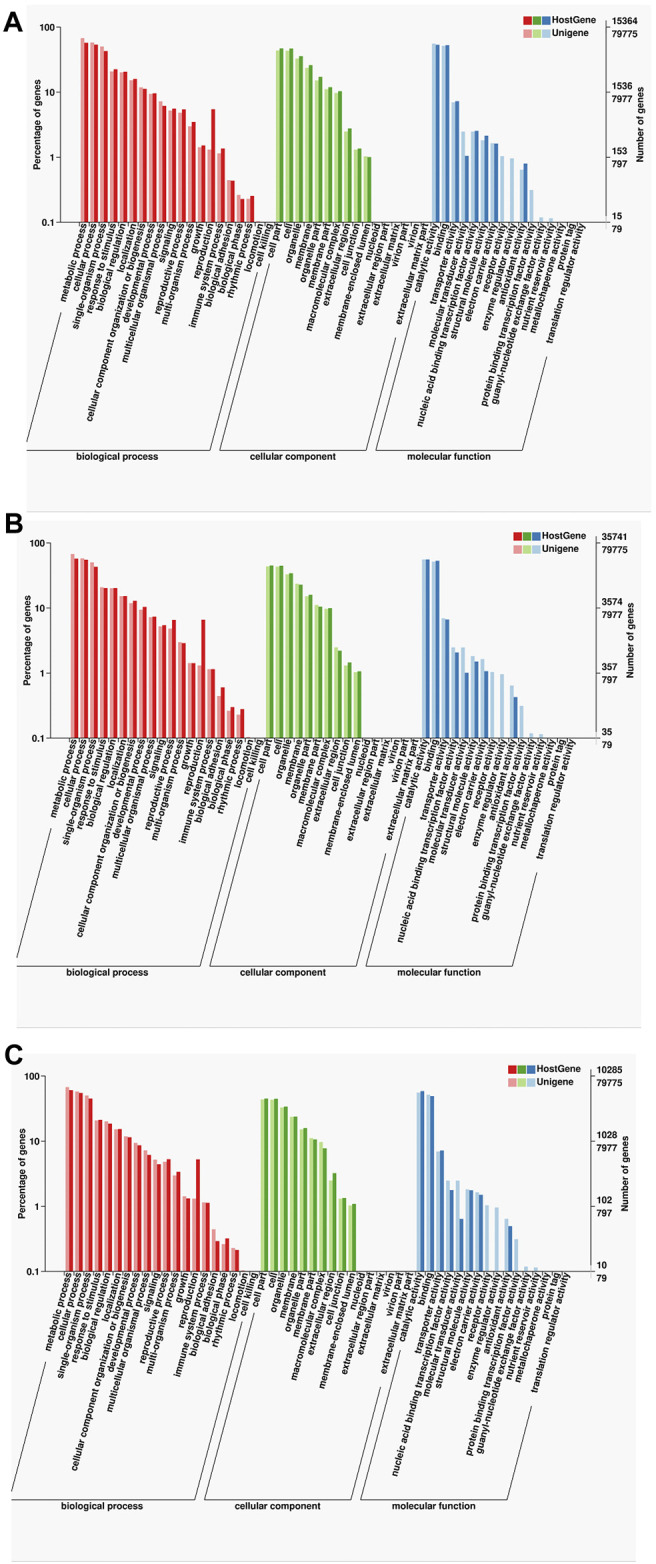
Statistics of the GO classification in two cultivars. **(A)** GO annotation classification statistics common in F01 and F02. **(B)** GO annotation classification statistics only in F01. **(C)** GO annotation classification statistics only in F02.

For further annotation analysis of gene functionality, the functions of specific and common isoforms in the two samples were analyzed systematically, indicating that although many of the isoforms in the two samples were different, the corresponding gene functions were similar. The GO analysis showed that many of the isoforms enriched in the following items: metabolic process, cellular single-organism, cell part, catalytic activity, and binding (Figure 6).

**FIGURE 7:**
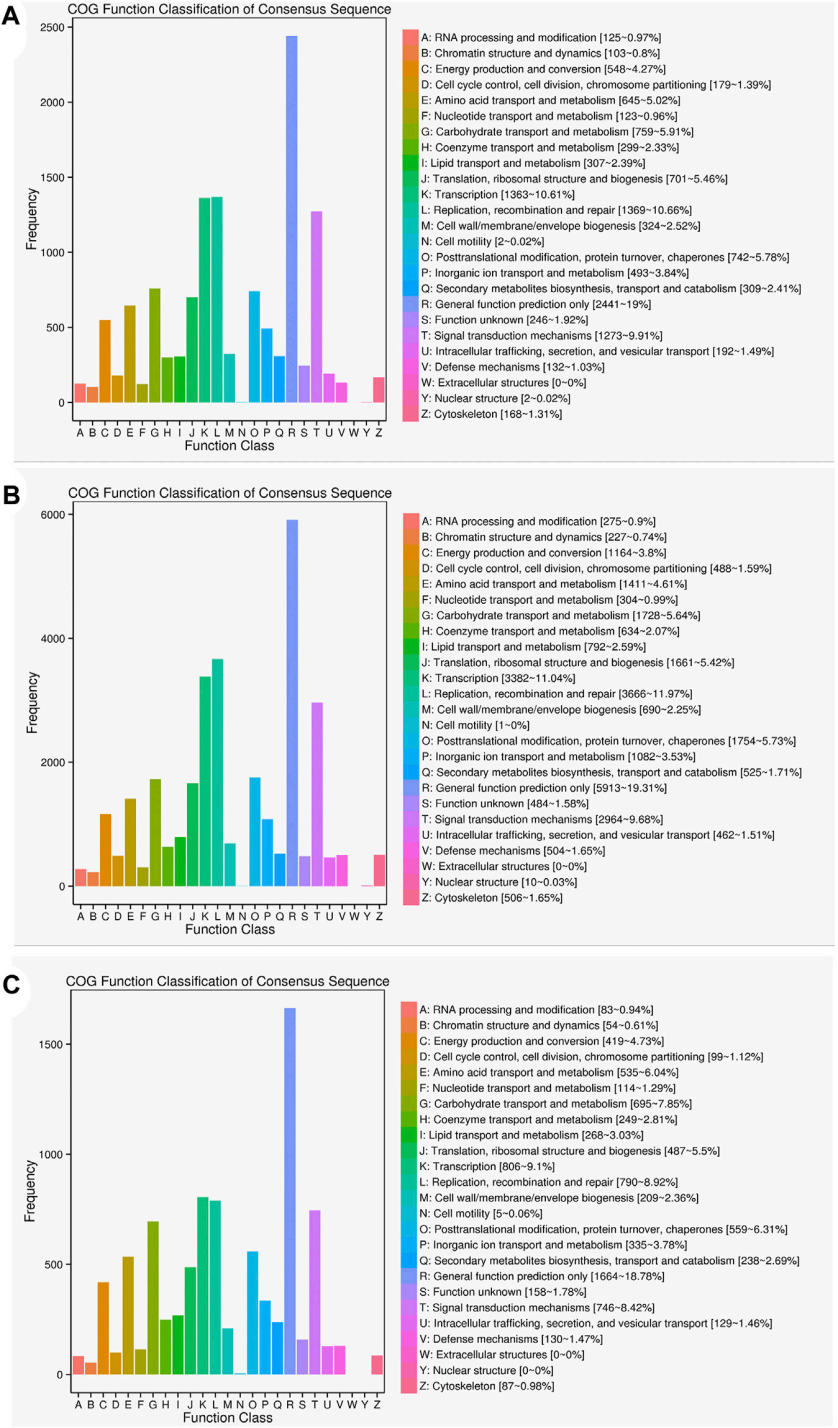
Statistics of the COG classification in two cultivars. **(A)** COG annotation classification statistics common in F01 and F02. **(B)** COG annotation classification statistics only in F01. **(C)** COG annotation classification statistics only in F02.

The results of the COG functional annotation analysis on the specific and common isoforms in Lm-type and normal castors were similar to that of the GO analysis, and the function of the isoforms remained consistent. The COG analysis indicated the most common gene functions in L (replication, recombination and repair) and R (general function prediction) (Figure 7).

### Genes Associated With Sex Expression and Reproduction in Castor

According to previous studies of sex determination between the monoecious and female *R. communis*, several subgroups genes were assumed to be putatively related to sex determination, such as auxin response factor, dynamin-2A, PCI domain containing protein, Xaa-Pro amino peptidase, ATP-binding protein, set domain protein, spermidine synthase, arginine/serine-rich splicing factor, eukaryotic translation initiation factor 2c, DNA (cytosine-5)-methyltransferase, s-adenosyl-methyltransferase, and acid phosphatase. In this study, many of these subgroups were identified in novel unigenes (Table 3). These genes were associated with hormone stimulus and participate in hormone-mediated signaling pathways, and also play a role in tissue and organ developmental processes.

**TABLE3:** Putative genes involved in sex determination of R. communis.

Gene name	Numbers in novel unigenes
Dynamin-2A	17
Auxin response factor	232
ATP-binding protein	3
Spermidine synthase	20
Arginine/serine-rich splicing factor	72
Acid phosphatase	123
Eukaryotic translation initiation factor 2c	124
Set domain protein	30
DNA (cytosine-5)-methyltransferase	23
S-adenosyl-methyltransferase	4

### Comparative Analysis of Differential Gene Expression Profiling

**FIGURE 8:**
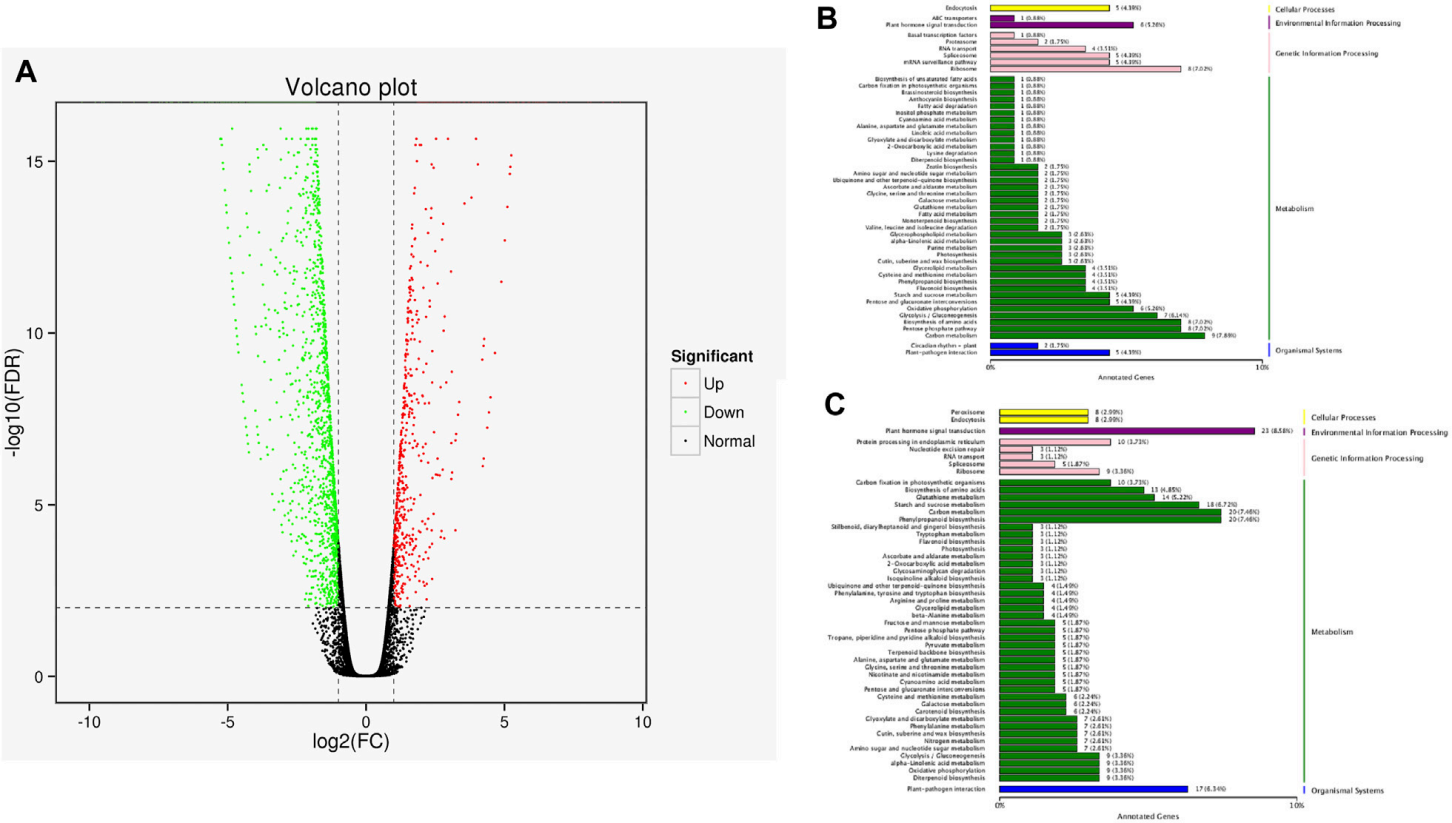
Statistics of DEGs and corresponding function in two cultivars. **(A)** Volcano plot of DEGs. The green dots represent down-regulated DEGs, the red dots represent up-regulated DEGs, and the black dots represent non-differentially expressed genes. **(B)** KEGG classification of up-regulated DEGs. **(C)** KEGG classification of down-regulated DEGs.

The full-length sequences were used as a reference genome, and the sequences from the short-read RNA sequencing were used to conduct a differential gene analysis. A total of 2,461 genes were found to be differentially expressed, with 655 up-regulated and 1806 down-regulated. These differentially expressed genes (both up-regulated genes and down-regulated genes) were classified according to their KEGG pathway (Figure 8). The results showed that up-regulated genes were classified as pathways of ribisome, carbon metabolism, pentose phosphate pathway, and biosynthesis of amino acids. The down-regulated genes were involved in plant hormone signal transduction, phenylpropanoid biosynthesis, carbon metabolism, and plant-pathogen interaction.

## Discussion

Alternative splicing was involved in phenotypic differences of Lm-type and normal castor cultivars.

In recent years, comparative transcriptome analyses have successfully revealed specific genes responsible for C4 photosynthesis in many grasses, including maize and switchgrass (Fan et al., 2019). Furthermore, recent studies of castor transcriptomes are mainly focused on gene expression from short-read RNA sequencing (Sood et al., 2014; Sturtevant et al., 2019) which cannot identify alternative gene splice forms (Fan et al., 2019). The development of full-length sequencing technology provides a span-new approach to study full-length sequences, alterative splicing, gene structures, and APA of RNA (Grabherr et al., 2011; Rhoads and Au 2015). We thus conducted a comprehensive comparative analysis for two cultivars using this method. In this work, a total of 76,068 and 44,223 non-redundant transcripts were obtained from the high-quality transcripts of Lm-type female strain and normal castor cultivars, respectively. Among these genes, 51,613 and 20,152 AS events were found in Lm-type female strain and normal castor, respectively, of which intron retention (IR) was the foremost AS event, with 62.14% and 55.94% in the two cultivars, respectively. Its confirmed gene expression and splicing levels may have a significant impact on the morphological and other phenotypic differences between the two cultivars (Wang et al., 2018). The alternative splicing of eukaryotic transcripts is a mechanism that enables cells to generate vast protein diversity from a limited number of genes (Baralle and Giudice 2017; Bush et al., 2017). The mechanism and outcomes of the alternative splicing of individual transcripts are well understood (Fan et al., 2019). Some studies find that AS regulation is independent or partially independent of transcriptional regulation (Siam et al., 2019) and implements great function at the early stage of the heat response (Keller et al., 2017; Siam et al., 2019), useful for future heat sensing and signaling studies (Fan et al., 2019). Our new findings about AS provide important information for facilitating castor genome annotation, and the full characterization of the castor transcriptome.

### Transcription Factors and lncRNAs Played Important Role in Phenotypic Differences of Lm-Type and Normal Castor Cultivars

As the key regulators of transcription, TFs play an important role in the physiological regulation of plants (Olsen et al., 2005; Zhou and Memelink 2016). Our results (Figure 3) suggested Rlk-pelle-dlsv, C3H, SNF2, and MYB-related transcription factors were the main types in the Lm-type cultivar. Transcription factors of rlk-pelle-dlsv, camk-camkl-chk1, MYB-related bHLH, and other types were mainly expressed in the normal castor cultivar. We speculate that the difference in TFs has a significant effect on the difference in morphology. Similarly, as the important regulator, the number of lncRNA was very different: there were 285 lincRNA, 58 antisense-lncRNA, 7 intronic-lncRNA, and 166 sense_lncRNA in the Lm-type cultivar, while 60, 22, 3, and 49 in the normal castor cultivar, respectively. Emerging work has revealed that many types of lncRNA regulate gene expression and have a great influence on genome stability in plants (Wang and Chekanova 2017; Sun et al., 2018). Studies on Arabidopsis show that lncRNA can serve as a molecular sponge and as a decoy, functioning in the regulation of transcription and silencing, particularly in RNA-directed DNA methylation, and in epigenetic regulation of flowering time (Zhao et al., 2018; Liu et al., 2019). Many plants reduce the expression of some lncRNAs to affect developmental phenotypes or molecular changes (Wang et al., 2014). We speculate that these regulators also played an important role in the growth and development of castor, and contribute significantly to phenotypic differences of Lm-type and normal cultivars.

### DEGs Implement a Significant Function in the Morphological Differences of the Two Cultivars

Using the full-length sequences as a reference genome, 2,461 differentially expressed genes were found, including 655 up-regulated genes and 1806 down-regulated genes, which was far more than in our previous RNA-seq transcriptome analysis (Fan et al., 2019). In this study, the functional analysis showed that proteins encoded by up-regulated genes (655) were classified to ribisome, carbon metabolism, pentose phosphate pathway, and biosynthesis of amino acids. Proteins encoded by down-regulated genes (1806) were attributed to plant hormone signal transduction, phenyl propanoid biosynthesis, carbon metabolism, and plant-pathogen interaction. We speculate that the differentially expressed genes were the main reason for the differences between the two castors, while the specific regulation mechanisms remain unclear.

## Conclusion

To the best of our knowledge, this study is the first large-scale comparative analysis of the transcriptome Lm-type and normal castor cultivars by single-molecule long-read sequencing. Comparative analysis of the isoforms, transcription factors, lncRNAs, and AS in the two cultivars was performed systematically. The gene annotation analysis showed that although the isoforms were diverse in the two cultivars, the implemented functions were similar. Many species-specific differences are mainly attributed to small effects at multiple loci, probably. However, differences in the expression of genes and alternative splicing events have a profound effect on the evolution of major morphological diversification for different individuals in the developmental processes. The new findings of this study provided invaluable information for facilitating genome annotation and the full characterization of the transcriptome of these two cultivars.

## Data Availability

The datasets presented in this study can be found in online repositories. The names of the repository/repositories and accession number(s) can be found below: https://www.ncbi.nlm.nih.gov/, SRR8662424; https://www.ncbi.nlm.nih.gov/, SRR8662425.
