# Supplementary figures and images for: A Comparative Transcriptional Landscape of Two Castor Cultivars Obtained by Single-Molecule Sequencing Comparative Analysis
Source: Front Genet. 2021 Oct 18;12:749340. doi: 10.3389/fgene.2021.749340 (PMC8558441; doi:10.3389/fgene.2021.749340)

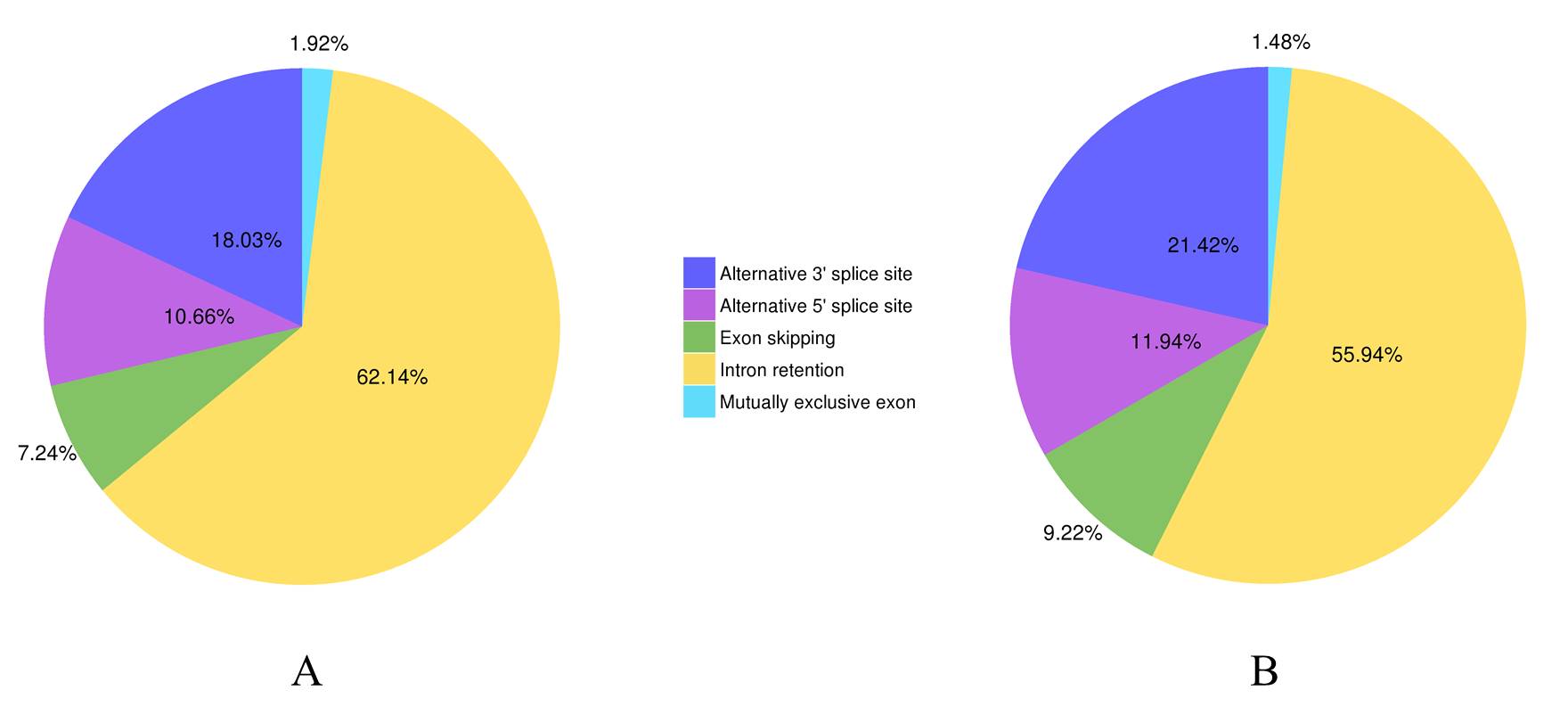

Supplement: Supplementary file 1 [file Figure2.JPEG]

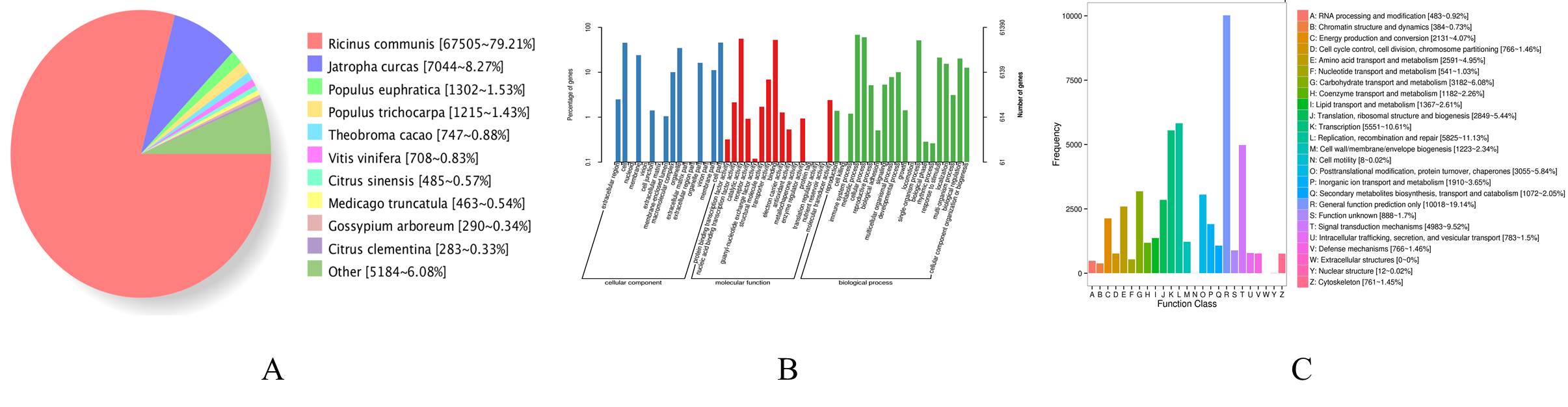

Supplement: Supplementary file 2 [file Figure5.JPEG]

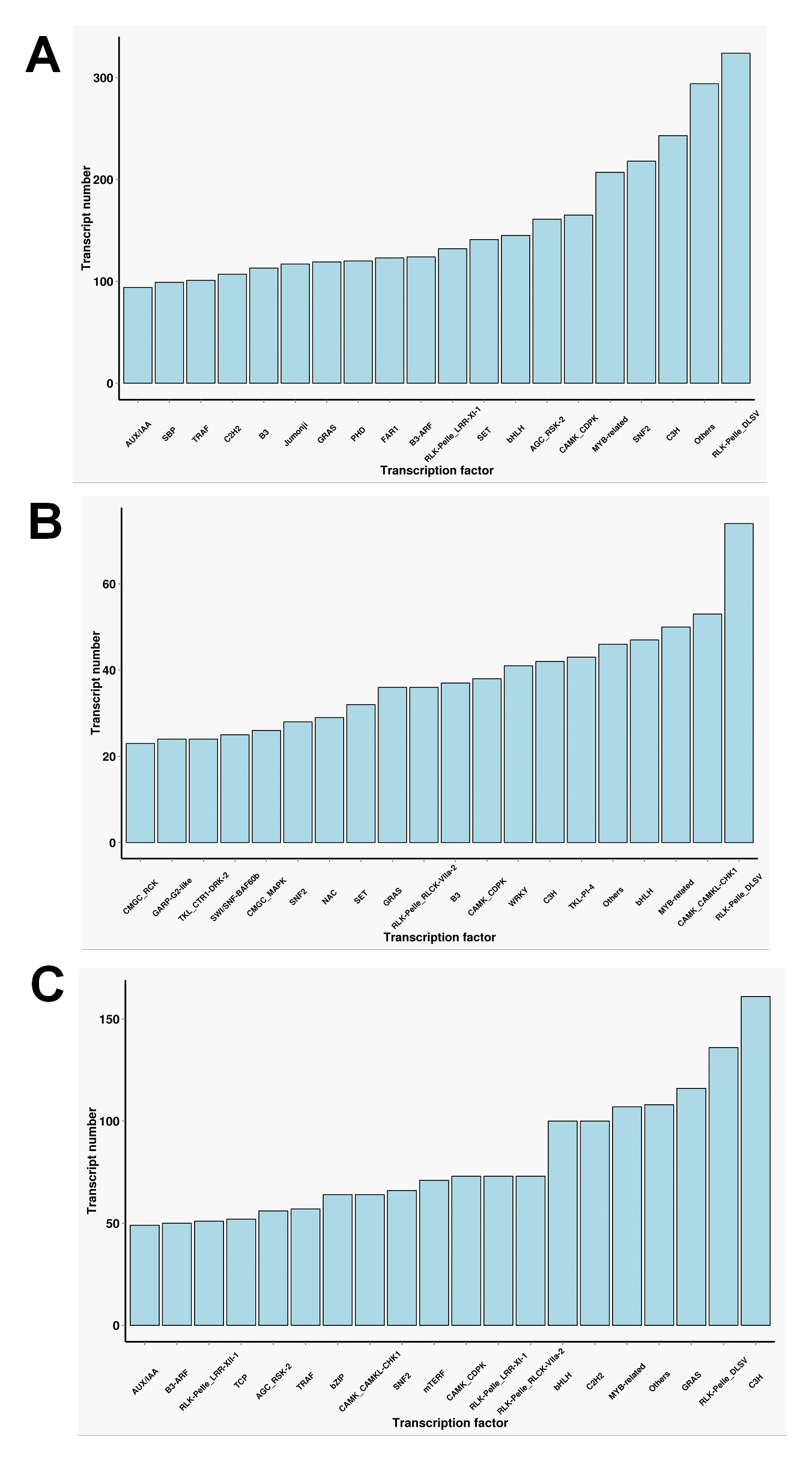

Supplement: Supplementary file 3 [file Figure3.JPEG]

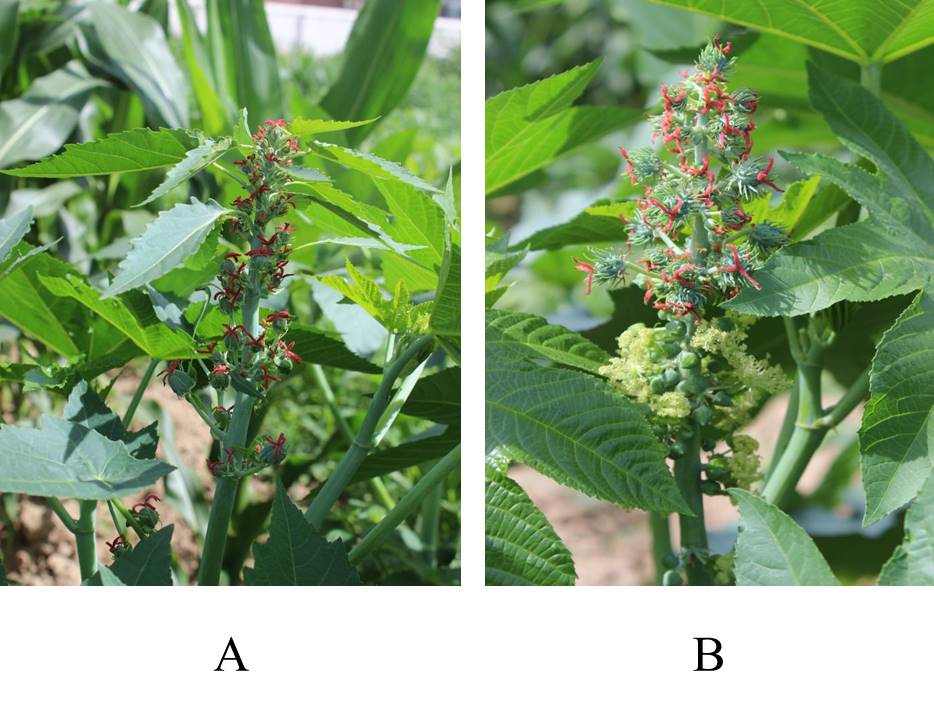

Supplement: Supplementary file 4 [file Figure1.JPEG]

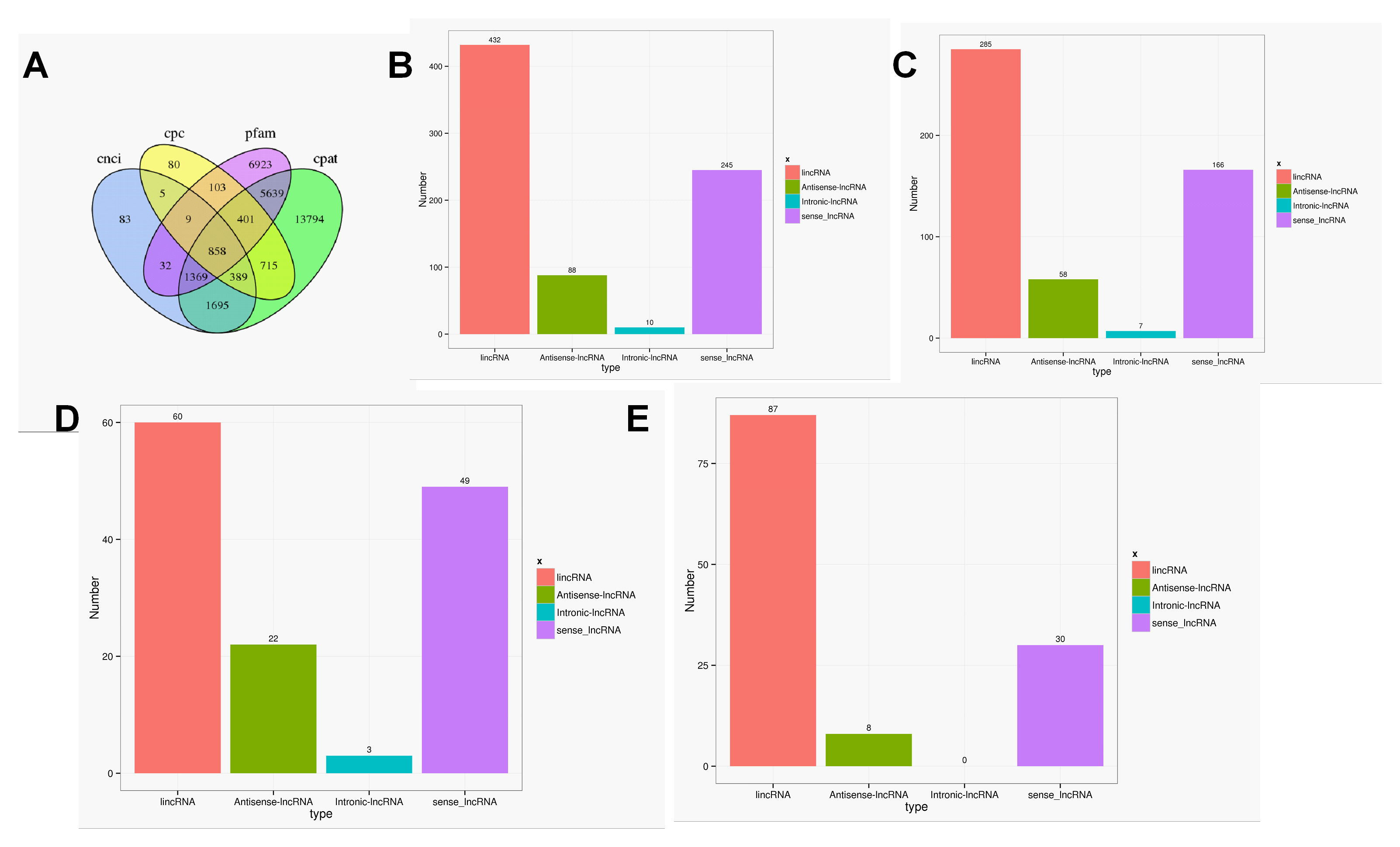

Supplement: Supplementary file 5 [file Figure4.JPEG]

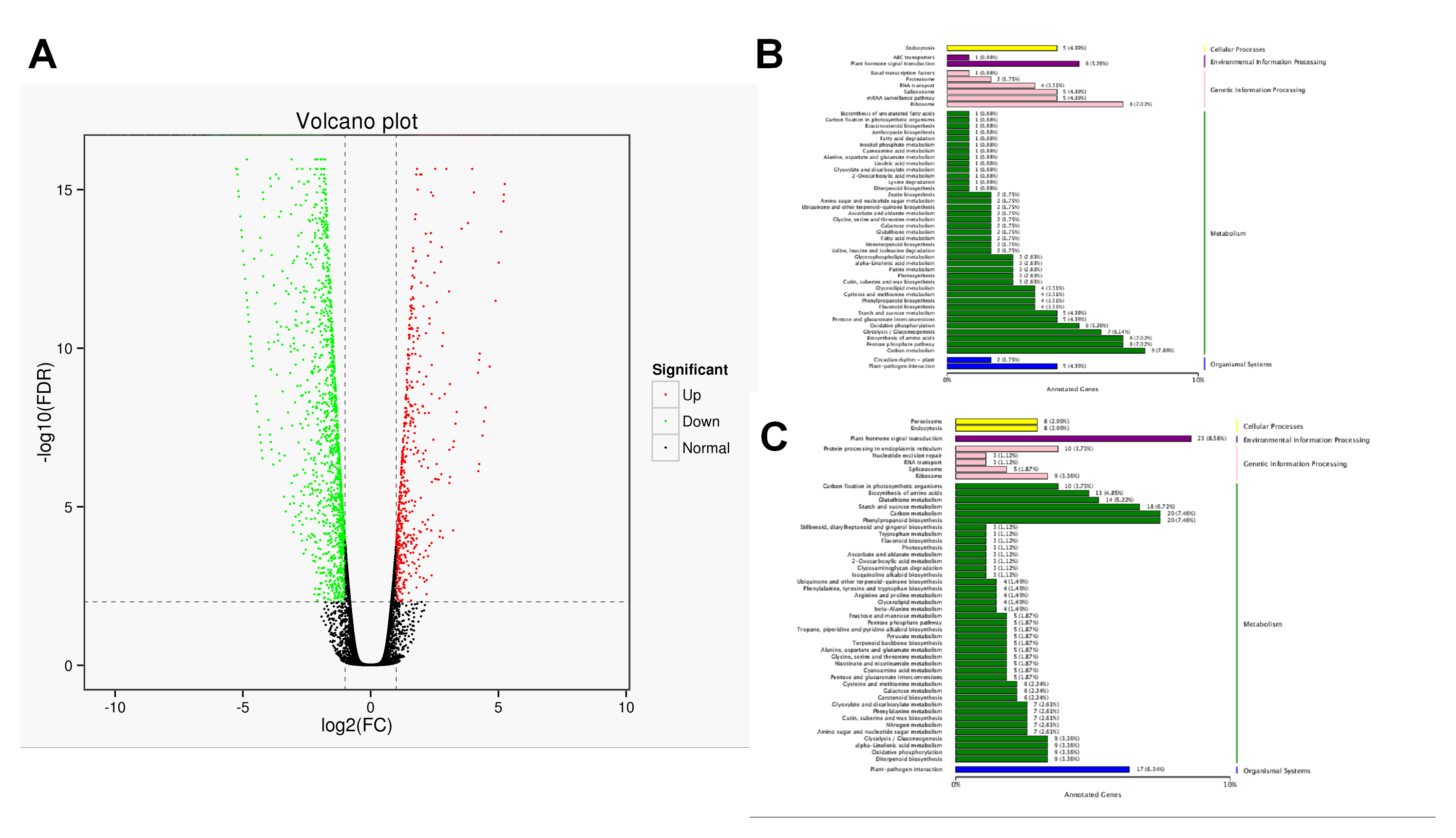

Supplement: Supplementary file 7 [file Figure8.JPEG]

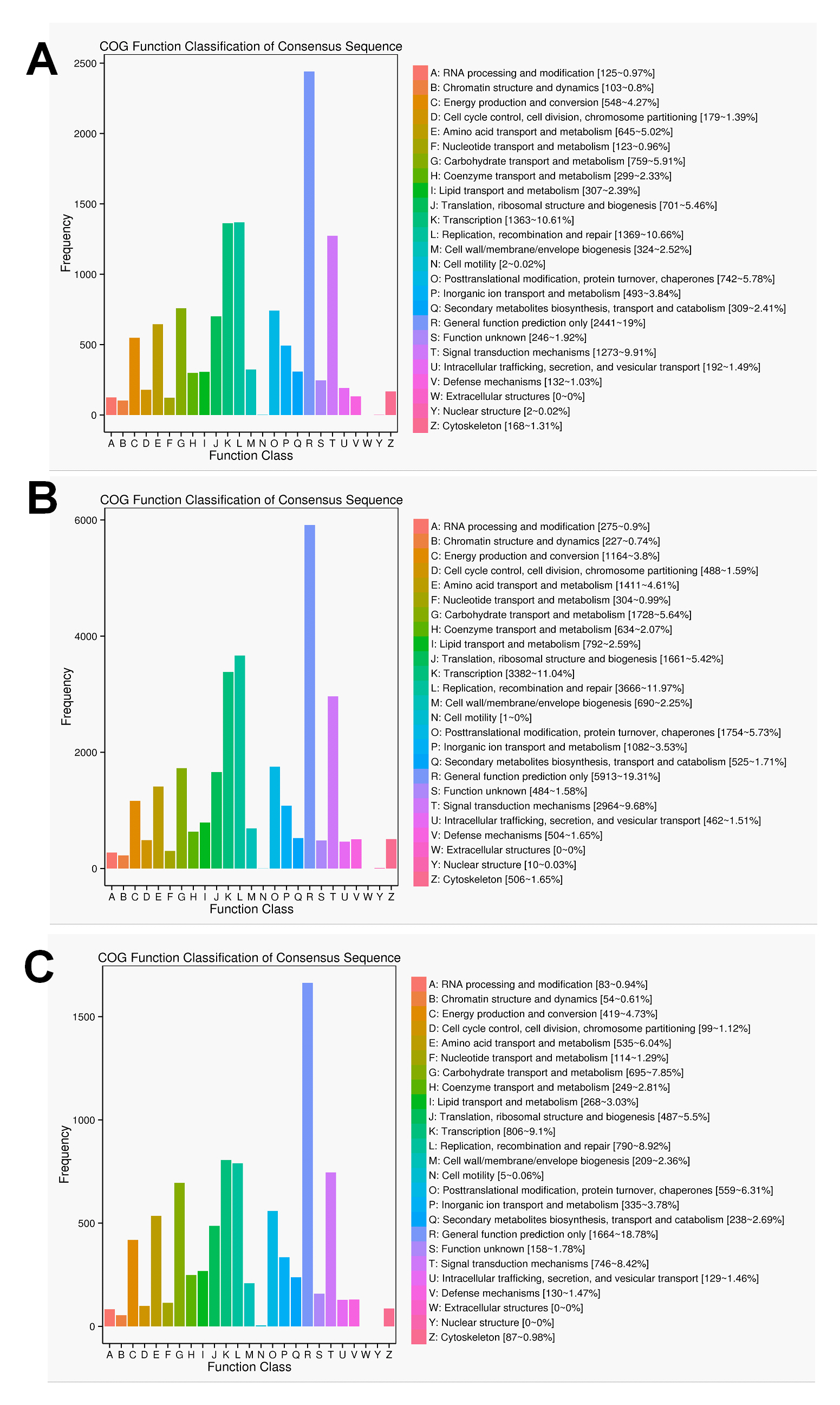

Supplement: Supplementary file 10 [file Figure7.JPEG]

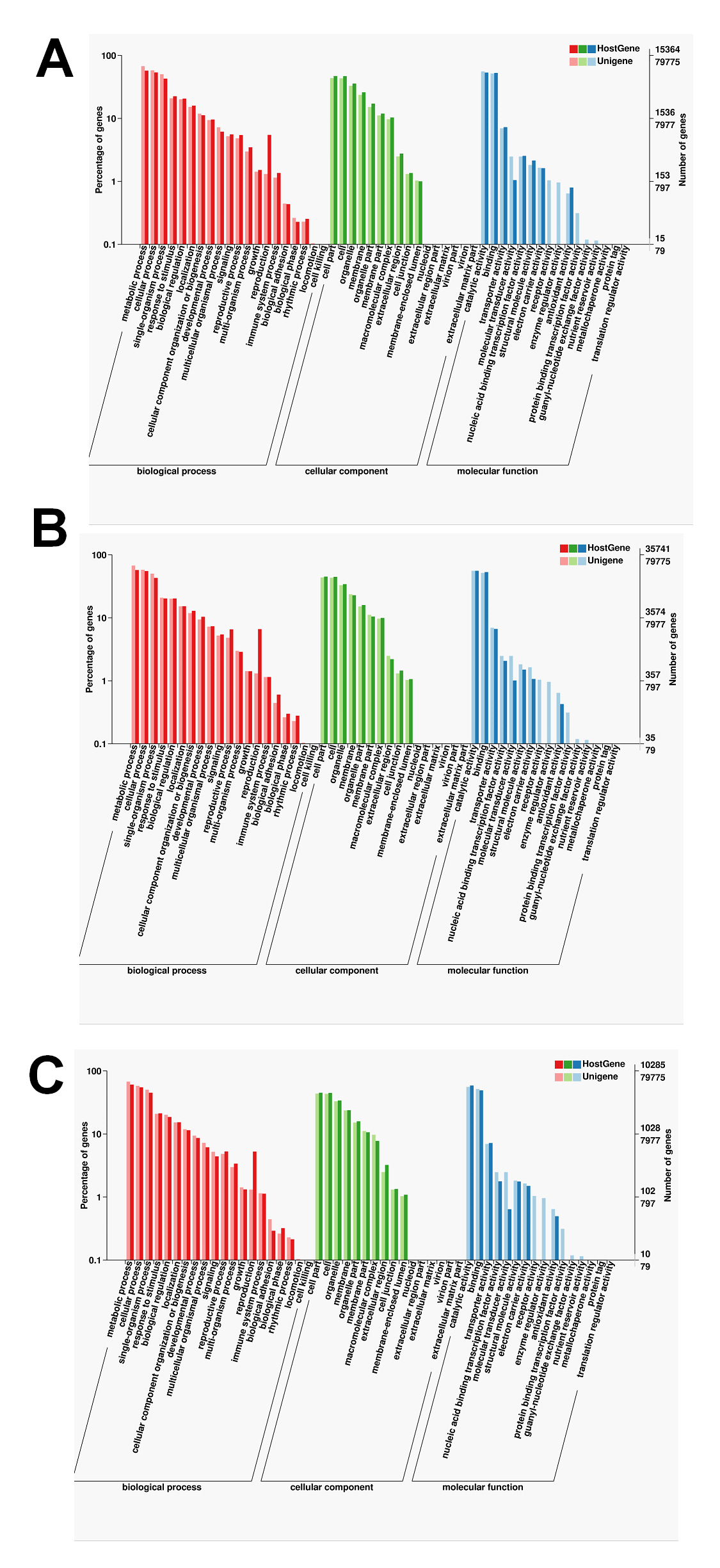

Supplement: Supplementary file 12 [file Figure6.JPEG]
