# Supplementary material for: A Comparative Transcriptional Landscape of Two Castor Cultivars Obtained by Single-Molecule Sequencing Comparative Analysis
Source: Front Genet. 2021 Oct 18;12:749340. doi: 10.3389/fgene.2021.749340 (PMC8558441; doi:10.3389/fgene.2021.749340)
Supplement: Supplementary file 6 [file Table7.DOCX]

Supplemental Table 3. The statistics of different AS events of Lm female strain and normal castor.

| AS_event | Lm female strain | Normal castor |
| --- | --- | --- |
| Mutually exclusive exon | 993 | 300 |
| Intron retention | 32073 | 11328 |
| Exon skipping | 3737 | 1868 |
| Alternative 5' splice site | 5504 | 2418 |
| Alternative 3' splice site | 9306 | 4338 |
