# Supplementary material for: A Comparative Transcriptional Landscape of Two Castor Cultivars Obtained by Single-Molecule Sequencing Comparative Analysis
Source: Front Genet. 2021 Oct 18;12:749340. doi: 10.3389/fgene.2021.749340 (PMC8558441; doi:10.3389/fgene.2021.749340)
Supplement: Supplementary file 8 [file Table1.DOC]

Table 1. The statistic results of ICE clustering.

| Samples | Number of consensus isoforms | Average consensus isoforms read length | Number of polished high-quality isoforms | Number of polished low-quality isoforms | Percent of polished high-quality isoforms (%) |
| --- | --- | --- | --- | --- | --- |
| F01 | 223,929 | 2,552 | 154,517 | 68,417 | 69.00% |
| F02 | 138,066 | 2,056 | 105,536 | 32,086 | 76.44% |
