# Supplementary material for: A Comparative Transcriptional Landscape of Two Castor Cultivars Obtained by Single-Molecule Sequencing Comparative Analysis
Source: Front Genet. 2021 Oct 18;12:749340. doi: 10.3389/fgene.2021.749340 (PMC8558441; doi:10.3389/fgene.2021.749340)
Supplement: Supplementary file 9 [file Table5.DOCX]

**Supplemental Table 1.Reads of insert (ROI) statistics.**

| Samples | Data Size(G) | cDNA size | Reads of Insert | Read Bases of Insert | Mean Read Length of Insert | Mean Read Quality of Insert | Mean Number of Passes |
| --- | --- | --- | --- | --- | --- | --- | --- |
| F01 | 26.25 | All | 647,205 | 1,565,216,652 | 2,418 | 0.95 | 16.00 |
| F02 | 16.38 | All | 328,497 | 657,361,379 | 2,001 | 0.97 | 23.00 |

Reads of insert: the number of ROI sequences; Read bases of insert: the total number of ROI bases; Mean read length of insert: average length of ROI; Mean read quality of insert: Quality value of ROI sequence; Mean number of passes: the mean sequencing depth of sequences in zero-mode wave.
