# Supplementary material for: A Comparative Transcriptional Landscape of Two Castor Cultivars Obtained by Single-Molecule Sequencing Comparative Analysis
Source: Front Genet. 2021 Oct 18;12:749340. doi: 10.3389/fgene.2021.749340 (PMC8558441; doi:10.3389/fgene.2021.749340)
Supplement: Supplementary file 11 [file Table3.DOCX]

Table 3. Putative genes involved in sex determination of *R. communis*.

| Gene name | Numbers in novel unigenes |
| --- | --- |
| Dynamin-2A | 17 |
| Auxin response factor | 232 |
| ATP-binding protein | 3 |
| Spermidine synthase | 20 |
| Arginine/serine-rich splicing factor | 72 |
| Acid phosphatase | 123 |
| Eukaryotic translation initiation factor 2c | 124 |
| Set domain protein | 30 |
| DNA (cytosine-5)-methyltransferase | 23 |
| S-adenosyl-methyltransferase | 4 |
