# Supplementary material for: A Comparative Transcriptional Landscape of Two Castor Cultivars Obtained by Single-Molecule Sequencing Comparative Analysis
Source: Front Genet. 2021 Oct 18;12:749340. doi: 10.3389/fgene.2021.749340 (PMC8558441; doi:10.3389/fgene.2021.749340)
Supplement: Supplementary file 13 [file Table6.DOCX]

Supplemental Table 2. Full-length sequences statistics.

| Samples | cDNA size | Reads of Insert | Number of five prime reads | Number of three prime reads | Number of poly-A reads | Number of filtered short reads | Number of non-full-length reads | Number of full-length reads | Number of full-length non-chimeric reads | Average full-length non-chimeric read length | Full-Length Percentage (FL %) | Artificial Concatemers（%） |
| --- | --- | --- | --- | --- | --- | --- | --- | --- | --- | --- | --- | --- |
| F01 | All | 647,205 | 527,979 | 531,986 | 510,652 | 16,754 | 174,353 | 456,098 | 448,217 | 2,374 | 70.47% | 1.73% |
| F02 | All | 328,497 | 294,357 | 293,222 | 283,600 | 4,315 | 60,404 | 263,778 | 258,645 | 1,931 | 80.30% | 1.95% |

Notes, cDNA size: insert fragment size of cDNA libraries; reads of insert: the number of reads of insert (ROI) sequences; Number of five prime reads: the number of ROI sequences containing 5′ primer; Number of three prime reads: the number of ROI sequences containing 3′ primer; Number of poly-A reads: the number of ROI sequences containing poly-A; Number of filtered short reads: the number of fltered ROI of <300 bp; Number of non-full-length reads: the number of non-full-length ROI; Number of full-length non-chimeric reads: the number of full-length non-chimeric ROI; Average full-length non-chimeric read length: average length of full-length non-chimeric sequence; Full-length percentage (FL %): the percentage of full-length sequence in ROI sequence; Artificial concatemers (%): the percentage of full length chimeric sequence in full-length sequence.
