# Supplementary material for: A Comparative Transcriptional Landscape of Two Castor Cultivars Obtained by Single-Molecule Sequencing Comparative Analysis
Source: Front Genet. 2021 Oct 18;12:749340. doi: 10.3389/fgene.2021.749340 (PMC8558441; doi:10.3389/fgene.2021.749340)
Supplement: Supplementary file 14 [file Table2.DOC]

Table 2 Statistics of annotations for genes of castor.

| Anno Database | Annotated Number |
| --- | --- |
| COG Annotation | 35,743 |
| GO Annotation | 61,390 |
| KEGG Annotation | 39,598 |
| KOG Annotation | 57,228 |
| Pfam Annotation | 63,089 |
| Swissprot Annotation | 62,336 |
| eggNOG Annotation | 83,826 |
| Nr Annotation | 85,286 |
| All Annotated | 85,322 |
